# Against Public‐Facing Religious Bio‐Restrictionism

**DOI:** 10.1111/bioe.70013

**Published:** 2025-06-25

**Authors:** Muralidharan Anantharaman

**Affiliations:** ^1^ Centre for Biomedical Ethics, Yong Loo Lin School of Medicine National University of Singapore Singapore Singapore

**Keywords:** bio‐restrictionism, public justification, reasonable disagreement, religious bioethics, respect, social conservatism

## Abstract

Recent calls to include religious bioethics on the table in policy and other public‐facing contexts have been made on the grounds of respect. This paper argues that these same considerations of respect point to an obligation to exclude religious bioethics from public‐facing contexts. This is because public‐facing religious bioethics is typically bio‐restrictionist in orientation and thus involves making demands on others that people could reasonably disagree with. At the same time, respect for persons grounds a public justification requirement according to which it is wrong to make moral demands on others that are subject to reasonable disagreement. Proponents of inclusion of these views are thereby committed to excluding such religious bio‐restrictionist views from public‐facing contexts.

## 1 Introduction

Recently, there have been calls to include religious points of view in bioethics, especially the more public‐facing kinds of bioethics. These calls tend to be grounded in the values of inclusivity and respect [1, 2, 3, 4, 5]. Consider Smith, who claims that bio‐restrictionist views “deserve a respectful hearing in the public square” [5, p. 12]. However, to the degree that including religious points of view in the public‐facing context involves, to some extent, making moral demands grounded in religious doctrines on those who could reasonably disagree, such inclusion would be disrespectful. As it turns out, calls to include religious bioethics in the public sphere are often driven by a desire to influence behaviour in a bio‐restrictionist direction [5, 6, 7, 8, 9, 10, 11]. Moreover, bio‐restrictionist views are subject to reasonable disagreement. For instance, people can reasonably disagree about the permissibility of abortion or euthanasia. Hence, insofar as proponents of religious bioethics are committed to respecting the reasonable diversity of moral outlooks, they should exercise restraint and take their own views off the table in public‐facing contexts. In this paper, I argue that these respect‐based arguments for the inclusion of bio‐restrictionist views in public‐facing contexts backfire. This is because the value of respect also grounds a duty to self‐exclude bioethical doctrines that are subject to reasonable disagreement.

My strategy in this paper is as follows: In Section 2, I will delineate what I mean by public‐facing religious bio‐restrictionism. I introduce what I mean by bio‐restrictionism, bio‐permissivism and reasonableness and then show why bio‐restrictionism is subject to reasonable disagreement. I then distinguish public‐facing bioethics from, on the one hand, inward‐looking bioethics, and on the other hand, scholarly bioethics. This delineation should make clear why public‐facing bio‐restrictionism involves issuing moral demands that are subject to reasonable disagreement. I then define what I mean by religious bioethics and explore the connection between religious bioethics and bio‐conservativism. In Section 3, I show why religious bioethicists who have appealed to the value of respect to justify the inclusion of their bio‐restrictionist views in public‐facing contexts are required to self‐exclude. I start by showing why certain appeals for inclusiveness made by religious bioethicists presuppose a duty of respect. Subsequently, I will rehearse an argument to the effect that respecting the diversity of reasonable moral views means refraining from making moral demands on others if those are subject to reasonable disagreement. Since including such religious bio‐restrictionist views in the public‐facing context involves making moral demands that are subject to reasonable disagreement, the duty of respect generates a duty for religious bio‐restrictionists to self‐exclude from such public‐facing contexts.

## 2 Public‐Facing Religious Bio‐Restrictionism

Before I spell out what is wrong with public‐facing religious bio‐restrictionism, it is worth demarcating our subject matter. In this section, I will try to address what I mean by the terms religious bioethics, public‐facing and bio‐restrictionism.

### 2.1 Bio‐Restrictionism, Bio‐Permissivism and Reasonable Disagreement

I will use the term bio‐restrictionism to describe socially conservative views^1^ in the field of bioethics. This includes not just opposition to human enhancement but also opposition to one or more of abortion, medical assistance in dying, assisted reproduction technologies like In‐Vitro fertilisation, paid surrogacy, In Vitro gametogenesis, gene editing, xeno‐transplantation, xeno‐gestation, research on embryonic stem cells and transgender care among others. By opposition, I mean that bio‐restrictionists judge the items on the above list as being morally wrong. Defined this way, one can be bio‐restrictionist about some items on this list, like medical assistance in dying, but not others like abortion or gene editing. One might also be bio‐restrictionist about a given item to various degrees. One might be opposed to abortion in all cases or perhaps only after some particular point of gestation.

We can contrast bio‐restrictionism with a family of bioethical views that are more permissive with regard to the items on the list. Let us call this family of views bio‐permissivism. As with bio‐restrictionism, people can be bio‐permissivist with respect to some items on the list, but not others and also to be bio‐permissivist about certain questions to different degrees. This last point might make it hard to distinguish at times between bio‐restrictionism and bio‐permissivism about a certain topic. For instance, is someone who believes that a medically unnecessary abortion is impermissible only after 32 weeks bio‐restrictionist or bio‐permissivist? Deciding where to draw the line on each of these topics is beyond the scope of this paper. Instead, we might simply treat those views that designate most instances of a certain kind of act to be impermissible as bio‐restrictionist. Thus, someone who considers abortion to be impermissible in most cases will be a bio‐restrictionist about abortion. Likewise, we may treat those views that designate most instances of a certain act as permissible to be bio‐permissivist. Someone who considers voluntary euthanasia to be permissible in most cases will be a bio‐permissivist.^2^

A core claim that I make in my objection to public‐facing religious bio‐restrictionism is that bio‐restrictionism is subject to reasonable disagreement. A proposition is subject to reasonable disagreement if it is possible for people to disagree about whether that proposition is true without thereby making a mistake in reasoning or missing some crucial piece of evidence.^3^ To say that bio‐restrictionism is subject to reasonable disagreement, I mean that for each of the listed topics, at least one of the bio‐permissivist views is reasonable. That is, someone^4^ who is morally committed and is not missing any decisive evidence could rationally endorse a bio‐permissivist view about any given topic. This is not to say that everyone who endorses it does so for good reasons. Rather, the claim is that it is possible^5^ for someone to endorse a bio‐permissivist position on a range of issues without lacking some crucial piece of available evidence [12] or making a mistake in reasoning.^6^ Neither is it to say that bio‐restrictionist views are unreasonable. Bio‐restrictionists and bio‐permissivists may just have different but equally good evidence for their beliefs.^7^

At least one version of bio‐permissivism about each item in the list is reasonable because at least one reasonable moral theory supports bio‐permissivist positions on these questions. To pick just one example, consider the principlist framework as outlined by Beauchamp and Childress [13]. The principlist framework, or at least some applications of the framework, straightforwardly supports bio‐permissivist positions on human enhancement, euthanasia, In Vitro fertilisation, paid surrogacy, In Vitro gametogenesis, germline gene editing, xeno‐transplantation and xeno‐gestation.

With regard to questions of euthanasia, paid surrogacy and xeno‐transplantation, considerations of autonomy count in favour of their permissibility. On many views, consent is a moral power that changes what permissions people have towards us [14, 15, 16]. Since in these cases, people can consent to the relevant acts, we can see how, on some views, they become permissible when freely consented to. One might think that worries about exploitation and hence considerations of justice would count against some of these, thus making things less straightforward. However, this would be true only under more egalitarian accounts of justice [17]. If one thinks that wealth inequalities are inherently unjust, or that agreements between people with vast differences in wealth and power are inherently suspect, then the fact that many people become surrogates because they need money might render the practice exploitative and hence impermissible. However, other, more libertarian accounts [18] of justice that deny that paid surrogacy is exploitative might also be reasonable. In addition, on some views, even if exploitative, removing options to earn money from those who are already desperate would be morally worse [17, 18].

It can also be reasonable to think that embryos have little moral weight, since they lack the biological pre‐requisites to reason or have subjective experiences. After all, no argument has conclusively shown that embryos have full moral personhood [19]. For instance, even quite sophisticated defences of the claim that human beings have moral status from the point of conception [20] are not quite decisive. On such views, human embryos are still beings that will, in the fullness of time, acquire full moral personhood. However, this does not contend with nonidentity problems [21]. Embryos that are aborted early or that are never implanted and remain in the petri dish will never develop into full moral persons. As such, these embryos would have little moral status. On such moral theories, early‐term abortion, IVF, IVG, surrogacy and embryonic stem cell research are permissible. Perhaps, there are, also reasonable, solutions to the nonidentity problem that will not have this result, but it is not clear that Harman's solution is irrational.

Lastly, even if not quite so straightforwardly, at least some reasonable ways of balancing the moral value of a foetus at a given stage of development and the risks and burdens to the pregnant person permit the termination of pregnancy at some of the later stages of pregnancy [22]. For instance, on Thomson's view, it is permissible to kill a person who is using (even if inadvertently) our body in a way that threatens our well‐being. If it is permissible to kill Thomson's violinist, then it should be permissible to kill foetuses even if foetuses count as full or nearly full moral persons. This makes at least some later term abortions permissible. There may be other views that deny that it is permissible to kill the violinist. On such views, one should not kill another even if at immense cost to one's welfare. However, there seems to be no decisive argument to show that those who endorse something closer to Thomson's view are making a mistake.

If all this is right, then bio‐restrictionism is subject to reasonable disagreement. Likewise, bio‐permissivism is also subject to reasonable disagreement. That said, we might notice a certain asymmetry once we isolate a particular bio‐permissivist view that I will call prime bio‐permissivism.

Recall that bio‐permissivism refers to a range of views that are on the more permissive ends of the spectrum of any given issue. It may be that not every bio‐permissive view is reasonable. There may, for instance, be decisive arguments against euthanising those who actively dissent or are against aborting healthy foetuses at 38 weeks of gestation if they can be safely delivered without risk to the mother. That is, on some issues, the most permissive views may very well be unreasonable. However, since not all bio‐permissive views are unreasonable, at least one must be reasonable. Consider the most permissive of the reasonable bio‐permissivist views. Let us call this view, whatever it is, prime bio‐permissivism. For this view, only its permissions are subject to reasonable disagreement. The requirements of prime bio‐permissivism are not subject to reasonable disagreement.

To illustrate, consider that it is almost certainly a requirement of prime bio‐permissivism about medical assistance in dying that we refrain from euthanising someone who does not consent to euthanasia and who actively dissents from this treatment. It is also a requirement of every other reasonable moral view that it is wrong to euthanise someone who does not consent and actively dissents. In order for there to be reasonable disagreement about this requirement, there would have to be a reasonable view on euthanasia that was even more permissive. Yet, by definition, there is no reasonable view that is more permissive than prime bio‐permissivism. Hence, there is no reasonable disagreement about the requirements of prime bio‐permissivism. This is not to say that this is where the line is to be drawn. Rather, the point is that whatever prime bio‐permissivism requires, other reasonable views also require.

There is, however, reasonable disagreement about the permissions of prime bio‐permissivism. This is because every other reasonable view is more restrictive than prime bio‐permissivism. To continue our example, consider that bio‐restrictionists would oppose euthanasia not just when there is no consent, but even when there is. Some intermediate positions may oppose euthanasia for those whose suffering is only mental. But arguably, the prime‐bio‐permissivist view would permit euthanasia in the case of purely mental suffering as well [23]. As such, the subject of reasonable disagreement is one in which the prime bio‐permissivist position is to permit it. This follows logically from the definition of prime bio‐permissivism as being the most permissive of the reasonable views on a question.

This question of whether permissions or requirements are subject to reasonable disagreement is important when a moral view is used as an interpersonal standard by which one may hold another to account. In this interpersonal or public‐facing context, insofar as a standard includes requirements subject to reasonable disagreement, that requirement is imposed on others. By contrast, this is not the case for permissions. This point will be developed more fully in the next section.

### 2.2 Public‐Facing Bioethics

To be sure, it is not bio‐restrictionism as such that I am objecting to. For all I know, some bio‐restrictionist views about some issues could be true. It is only bio‐restrictionism in the public‐facing context that I object to. This, of course, raises the question as to what the public‐facing context is. The public‐facing context refers to those situations involving second‐personal address [24] that involves the actual exercise of power or at least the attempt to do so. Situations involving second‐personal address can be contrasted with first‐personal (which is more about self‐direction) and third‐personal address (which is a more detached perspective). As an example of the second‐personal address, think of those cases where one person makes a demand on, provides advice or suggestions to others or gives them permission. We can contrast this with the first‐personal case, where someone might use certain moral or religious doctrines to make a decision about their own behaviour. For instance, a patient who refuses a blood transfusion on the basis of her religious beliefs may be invoking religious bioethics, but since this is used to direct her own actions, this is a first‐personal case and not public‐facing. The paradigm of third‐personal case involves scholarly bioethics,^8^ in which we explore what a certain moral or religious viewpoint says about a certain topic in bioethics or see where an argument leads us. Here, there is no implication that one has to abide by any requirements. One is free to reject the underlying moral or religious doctrine at least insofar as accepting the underlying doctrine is not itself *required* by rationality.

By contrast, the paradigmatic public‐facing cases are ones involving advocacy, activism, regulations and guidelines. Another feature of this public‐facing bioethics is that in the public‐facing context, making moral demands on others is an attempt to exercise power, whether soft or hard, on those who disagree. Moral demands are directives to do (or not do) something where the issuer of the directive intends that act (or omission) is morally required for the recipient of said directive. Public policies and regulations are a clear case where hard or coercive power is exercised. When activists and advocates seek to implement or change policies or regulations, this may involve calling upon the state to coercively change certain people's behaviour. Insofar as bioethics has a practical remit [25, 26], it will be involved in such activities. For example, activists might instead protest outside an abortion clinic and berate the patients entering the clinic or protest outside a lab that uses stem cells for research to use the soft power of social pressure to get patients and researchers to do as the activists want despite the former's misgivings. Somewhat in between paradigmatic cases of hard and soft power are ones involving the internal rules and decisions of institutions. For instance, Institutional Review Board^9^ (IRB) decisions [12] rely on more than just social pressure. Biomedical research that is conducted without IRB approval may fall afoul of the law, be denied funding or licencing or at least may be rejected by journals. Yet, IRBs do not merely enforce the law; they also have discretion in interpreting legal requirements and may even appeal to standards that go beyond what the law requires. Either way, making moral demands in the second‐personal stance involves an exercise of power that is potentially problematic.

The attempt to exercise power is an important component of the public‐facing context, as there might be instances of second‐personal address that do not involve an exercise of power or an attempt to do so. For instance, an anti‐abortion activist, Alice, could make a moral demand that a pregnant person, Polly, refrain from having an abortion. Instead of using social pressure to accomplish her goals, Alice instead tries to make the best arguments that she can in favour of her claim. When Polly remains unconvinced by her arguments, Alice backs off and does not insist. Even if this counts as an instance of second‐personal address, it does not count as public‐facing in the relevant sense.^10^ Care should be taken, however, that social pressure is not inadvertently exerted. One person telling another may exert only a negligible amount of social pressure, but a thousand people doing the same exerts significant pressure. A single signature on a petition does not mean much, but a million signatures can have a significant impact on people's behaviour.

The fact that the requirements of bio‐restrictionism are subject to reasonable disagreement means that public‐facing bio‐restrictionism would involve making moral demands on others who may reasonably disagree in a way that involves an attempt to exercise some power over them. This, as I will argue in Section 3, is morally objectionable. By contrast, since none of the *requirements* of prime bio‐permissivism are subject to reasonable disagreement, public‐facing prime bio‐permissivism does not involve issuing moral demands on others who reasonably disagree with those demands. To be clear, since the permissions of prime bio‐permissivism are subject to reasonable disagreement, prime bio‐permissivism in the public‐facing context would involve attempting to give social licence to acts, the permissibility of which people may reasonably disagree about.

### 2.3 Religious Bioethics

Religious bioethics is significant to public‐facing bio‐restrictionism for three reasons. First, religious doctrines are often used to ground bio‐restrictionist conclusions. Second, arguments for the inclusion of religious bioethical views in the public sphere have been made with the intention of shifting public policy or social norms in a bio‐restrictionist direction Third, the argument from adequacy (which I will define later) is made primarily by religious bioethicists.

Let me explain what I mean by these terms. By religious bioethics, I mean bioethical theories that at least partly depend on theological, mythological or scriptural claims.^11^ By contrast, religion in bioethics includes not only religious bioethics but also other sorts of cases. For instance, taking into account a patient's religious beliefs when accounting for their best interests in a given case is clearly a case of religion in bioethics, but reasons for doing so need not be grounded in any given religious doctrine. When a Jehovah's Witness patient refuses a blood transfusion or when a Muslim patient worries about how a stoma would affect their daily prayer, while we may need to be sensitive to the nuances of their religious beliefs in order to treat the patient ethically, our reasons for doing so need not be grounded in their religious doctrines. Instead, considerations of beneficence and autonomy are what ground our treatment of the patient in these cases. Secular bioethics can be sensitive to local cultural and religious differences.

Defined as such, religious bioethics need not be bio‐restrictionist and bio‐restrictionism need not be religious. After all, certain kinds of Aristotelian views as well as Confucian bioethics can have bio‐restrictionist implications [27, 28] without necessarily counting as religious. Likewise, liberal interpretations of religions when applied in the bioethical context may have more bio‐permissivist implications. For example, some Christians accept euthanasia on the grounds that it is merciful to allow a patient to die when they would otherwise endure intolerable suffering.

However, even if religion is neither necessary nor sufficient for bio‐restrictionism, there is a systematic connection that makes the question of religious bioethics relevant to this paper. To see the systematic connection, see first that religious bioethics is *often* bio‐restrictionist. In particular, *public‐facing* religious bioethics is almost invariably bio‐restrictionist [29]. We do not, for instance, see religious bio‐permissivists arguing that their religious point of view should be taken seriously in public‐facing contexts and hence move the Overton window on public policy and public sentiment. By contrast, we can easily produce examples of religious bio‐restrictionists [4, 6, 8, 9, 11, 30, 31] doing the same. When religious bioethicists complain about mainstream secular bioethics, the complaint can often be about secular bioethics being too liberal or permissive on a range of matters. Chia, for instance, claims that ‘by its openness to transcendence, the Church can also avoid the secularism and anthropocentrism that are so prevalent in modern culture’ [6, p. xv]. Plausibly, insofar as the dominant secular bioethics paradigm tends towards bio‐permissivism, religious bio‐permissivists have few complaints about the current paradigm. Religious bio‐restrictionists, on the other hand, might see some of their concerns systematically being given less weight than they believe warranted.

This is, of course, not merely happenstance. One way to understand social conservatism in bioethics is to see such views as driven by a concern with purity or sacredness [32] among other considerations.^12^ Sacredness/purity is not a single value that has concrete implications. Rather, claims are made to certain things being sacred and further, this fact requiring certain responses and forbidding others. However, such claims can only be justified from the perspective of certain religious traditions. To see what I mean, consider Sandel's argument against human enhancement:

The problem is not the drift to mechanism but the drive to mastery. And what the drive to mastery misses and may even destroy is an appreciation of the gifted character of human powers and achievements.

[10, p. 27]

Sandel is considering what precisely makes human enhancement and genetic engineering wrong and is ruling out various candidate explanations. One of the candidate explanations that he rules out here is that they ‘undermine effort and erode human agency’ and make us more robotic [10, p. 26]. Sandel's own diagnosis is that such technologies offer us too much agency. On his view, the very ends that are being sought are morally problematic because those ends are constitutively about seeking to remake human nature for our own ends. In other words, human powers are sacred and may not be upgraded and enhanced willy nilly. Indeed, Sandel's distaste for remaking nature or human nature echoes Abrahamic injunctions against playing God. Sandel acknowledges that the claim has a quasi‐religious character to it and tries to appeal to various intuitions to bolster his claim. However, such bare appeals to intuition have limited probity. Without the theoretical heft of religious doctrine, speaking about the gifted character of human powers is just so much airy speculation.

Sandel's point is precisely that our abilities are not our own to do with as we will, constrained only by duties to not harm or violate others' rights. If our abilities are gifts, then there must be a gift giver, or rather a gift Giver [33, 34]. Consider the logic of Sandel's argument. If our abilities are merely a gift from our parents, then while we could not enhance ourselves willy nilly, our parents can enhance us. Likewise, if my abilities are a gift from the polis, then we could decide who gets what enhancements by a democratic procedure. Any other source of the gift other than God still permits somebody or some collection of persons to enhance our abilities. The only way Sandel can maintain that the giftedness of our abilities forbids enhancement by anyone is by attributing the gift to God. Notably, this requires a very specific theological claim. If, for instance, one was Hindu or Buddhist and endorsed the doctrine of reincarnation, then the abilities that I am born with are merely the products of my karma from my previous life. That is, they would be the consequences of my actions in previous lives. The latter picture can easily allow enhancement because getting an enhanced body in this life would still be the result of the karma from previous lives. Sandel's claim about the gifted character of human abilities thus requires certain religious doctrines. Perhaps a number of different religions would be consistent with the giftedness claim, but it is not trivially consistent with the doctrines of every major religion. And it is certainly not likely to be justified by a purely secular moral framework.

Likewise, we could say something similar about bio‐restrictionist positions on other issues. Consider, for instance, Kass's defence of repugnance. The feeling of repugnance is explicitly, on his account, an emotional response to violations of sacredness or purity norms [35]. Kass himself doubts the ability of secular arguments to fully defend the judgement that the acts judged as repugnant are wrong. His attempts at defending such claims are weak [36]. For instance, his objections to reproductive cloning and genetic screening of embryos among other things are premised on claims like ‘the inherent procreative teleology of sexuality’ [35, p. 18] or on ‘male and female complementarity’ [35, p. 18].^13^ Ostensibly, such arguments make no explicit reference to religious doctrines. However, without accepting some controversial religious or metaphysical doctrine, it is hard to see what rational purchase such arguments may have. For instance, why should we think that sexuality has only one inherent procreative *telos*? The desires that arise from a cis‐gendered heterosexual person's sexuality can be satisfied by procreative acts. However, they could also be satisfied by non‐procreative acts. For others, sexual desires will typically be satisfied only by acts that are not procreative. It may be that sexual urges evolved in vertebrates because it contained a fitness advantage by increasing the likelihood that such animals would engage in procreative acts. However, while scientists might use teleological language to describe sexuality, this is merely shorthand for the rather mundane fact that sexuality is a trait that confers a certain kind of fitness advantage under certain conditions [37, 38]. The natural facts, as such, do not seem to suggest that sexuality has exactly the kind of purpose that Kass needs in order for his argument to succeed. While Kass needs heavily laden metaphysical claims to ground his sacredness norms, secular arguments cannot get him there. He needs the theoretical heft of religious doctrine to ground the kinds of metaphysical claims that would vindicate the sacredness intuitions he thinks are important. Perhaps there are some questions for which the bio‐restrictionist position can be defended without recourse to considerations of sacredness, but it should be clear that most of the dialectical force in favour of bio‐restrictionist positions has some religious source.^14^

Relatedly, a key target in this article is the kind of argumentative strategy that religious bioethicists^15^ employ: They employ, what I call, the argument from adequacy. Roughly, the strategy is to argue or at least imply that religious bioethics should be included in the public‐facing context because it is *epistemically adequate*. This argument will be examined in Section 3 in greater detail, where I will argue that it also commits religious bioethicists to a public justification requirement.

Notably, the argument from adequacy is not a religious argument. The peddling of this argument by certain religious bio‐restrictionists is merely a contingent matter. Even if contingent, it is not merely by chance. There is a reason why religious ethicists do not simply offer what they see as the best reasons for their conclusions and proceed from there. The argument from adequacy evinces a defensive posture that makes sense in a post‐Enlightenment context where religious belief is often regarded as irrational [39]. Even if not regarded as outright irrational, the common sentiment is that there is no hope of establishing by argument any one particular religion's doctrines as true beyond any reasonable doubt. Among philosophers, at least, there is significant agreement that we can access moral truths without recourse to religion [40]. This explains the contrast where religious bioethicists feel the need to establish the bonafides of their approach while secular ethicists feel free to simply make their argument.

Taking stock, even though there is no necessary connection between bio‐restrictionism and religious bioethics, religious bioethicists pushing for inclusion in the public‐facing context are predominantly bio‐restrictionist^16^ and this correlation is not merely accidental. Therefore, the inclusion of religious bioethics in the public‐facing context will result in public‐facing bio‐restrictionism whose requirements, as we saw, are subject to reasonable disagreement.

To be sure, demands for inclusion of religious bioethics are not always demands for a particular religious view to dictate how everyone should behave. Certainly, activists working from a particular religious viewpoint will make claims on others on the basis of their own religious viewpoint. However, recent calls for inclusion of religious bioethics especially in policy and ethics boards settings are calls for the inclusion of such views as one among other viewpoints on the table [3, 41]. Any meaningful inclusion, however, will still involve some departure from prime bio‐permissivism. As such, some of the requirements of the resultant view would be subject to reasonable disagreement.

For instance, suppose that the prime bio‐permissivist view would permit most kinds of embryonic stem‐cell research, while on a certain religious view, embryonic stem cell research should be completely forbidden. Meaningfully including that religious view on the table in a policy‐making setting would mean that some kinds of embryonic stem cell research would be forbidden, while others would be permitted. Even with such a compromise, the moral demands on researchers that are thereby made are subject to reasonable disagreement. After all, by hypothesis, prime bio‐permissivism is a reasonable view. Thus, any inclusion of religious bio‐restrictionist views in the public‐facing context will result in a violation of a public justification requirement according to which moral demands, in the public‐facing context, may be permissibly made only if no one could reasonably disagree with those demands.

Moreover, as I will argue in the next section, the argument that they feel compelled to give for inclusion in the public‐facing context commits them to a public justification requirement. If this is right, then given that the requirements of bio‐restrictionism are subject to reasonable disagreement, religious bioethicists have a duty to self‐exclude their bio‐restrictionist views from public‐facing contexts.

## 3 Respect, Inclusion and the Public Justification Requirement

In this section, I will argue not just that moral demands, especially in a public‐facing context, are subject to a public justification requirement, but also that proponents of the inclusion of religious bioethics in public‐facing settings are also committed to affirming such a public justification requirement. If my arguments succeed, then by their own lights, proponents of public‐facing religious bio‐restrictionism have a duty of restraint to only make publicly justifiable demands on others.^17^ If this is right, even the compromise generated by religious bioethics being only one among the different voices at the table will be impermissible.

The key motivation for including religious bioethics in the public‐facing context is one of respect [3, 5]. To see why, we must examine the argument from adequacy. We will look at two exemplars of the kind of argument that I am talking about, but others have made similar moves in defending religious bioethics [8, 9, 11, 30, 31, 42, 43]. Camosy and Cahill, in defending the role of religious bioethics in the public sphere, argue that the secular sphere is not neutral and that liberalism is just one moral tradition among others. There is hence no reason why religious doctrines should be excluded from the public sphere [4, p. 38, 42, pp. 158–159]. Chia claims that religion should be included because it can make significant contributions to public discourse in bioethics [6, p. xv]. What is common to these arguments is the implicit commitment to the claim that religious bioethics should be included because such theories are epistemically adequate. In Cahill's argument, liberalism being non‐neutral would be irrelevant if religious bioethics were to be poorly justified. Likewise, for Chia, religion could not contribute to public debates in a meaningful sense if religious doctrines were irrational to believe or rational only for those who lack some crucial piece of evidence. Let us call the proposition that, a given religious doctrine can be rationally believed by someone who is not lacking some decisive piece of evidence (i.e., that it is reasonable), the epistemic Adequacy claim.

These arguments also presuppose that a moral doctrine being epistemically adequate in the above sense is sufficient for inclusion. For Cahill, this seems implicit in her denial of liberal neutrality. Since neither liberalism nor any other doctrine is neutral in the relevant sense, being neutral cannot be a necessary condition for inclusion. Neither does Cahill seem to think that there is any other criterion that determines whether a moral theory or tradition should be included in the public sphere. Likewise, while Chia focuses on the profoundness and depth of the Christian tradition as reasons to include Christianity in public bioethical debates, he does not propose to exclude any view for lacking profoundness and depth. If, on their view, being epistemically adequate for belief or reasonable is not sufficient, their arguments would have taken a different tenor. They would have tried to show how their own view was superior to rival views that ought to be excluded. Since they did not, we can safely conclude that on their view, being reasonable is sufficient for inclusion in the public sphere. Let us call this the Sufficiency claim. These two claims combine to constitute the argument from adequacy. We can represent the argument schematically as such^18^:

1. Bioethical view, V, based on a certain religious doctrine is epistemically adequate for belief/reasonable. (Adequacy)
2. If a moral doctrine is adequate, it should not be excluded from the public sphere. (Sufficiency)
3. V should not be excluded from the public sphere.

For those who accept Sufficiency, the only reason to exclude a bioethical view from the public sphere is if it is not adequate. Hence, on this view, to exclude religious bioethics is to treat religious belief as unreasonable when it is not. This, arguably, is disrespectful to religion [30]. We might also think of this as a failure to respect persons because treating a reasonable view as unreasonable involves a failure to recognise that an equal moral agent could, without making a mistake or lacking crucial information, endorse that view. It can also be seen as disrespectful if religious bioethics is singled out to be excluded but other nonreligious bioethical theories that are epistemically on a par with religious theories are not excluded. While there is little doubt that many who oppose religious bioethics in the public sphere do so because they regard all religious bioethics to be inadequate, I will do so by instead rejecting Sufficiency.

Recall that the objection to exclusion was that exclusion of religious bioethics disrespects religious points of view by (falsely) implying that they are epistemically deficient in some way or by failing to respect the other person's equal moral status. By the same measure, making moral demands in public‐facing contexts that are subject to reasonable disagreement is disrespectful because it falsely implies that alternate viewpoints are epistemically deficient and fails to recognise their equal moral status.

To see why, let us consider the conditions under which we might make moral demands on others. Our practice of making moral demands on one another can be divided into good and bad kinds of cases. As noted above, making moral demands in the public‐facing context involves an attempt to exercise power over others. In the bad kind of case, this amounts to pushing people around [12, 44], or at least attempting to. In the good kind of case, making moral demands on others serves to enable social coordination and remind people of what morality requires of them.

The bad case occurs when the demand is made on someone who could reasonably disagree. This is because when the disagreement is reasonable, everyone could continue to disagree even if they reasoned impeccably and nobody's evidential situation gives them undefeated access to the truth. That is, everyone is evidentially on a par with each other. In such cases, demanding that others do something as if we had superior access to the moral truth [12] would falsely impute that the other person's beliefs were epistemically deficient. This is the same kind of disrespect that proponents of religious bioethics were worried about.

In addition, it is disrespectful to the recipient of the demand because it supposes that she is obligated to follow our commands simply because we have commanded it. This point is important because one might object that we ordinarily do not regard making moral demands or even coercing those who reasonably disagree as disrespectful. Yet, to suppose that someone is obligated to follow our commands simply because we have commanded it is disrespectful, as it fundamentally fails to recognise the other person's status as a moral equal. No one is ever the natural slave of somebody else. However, if someone is obligated to follow our commands simply because we have commanded it, they would be our natural slaves. In the case where the demander's belief is false, it is easy to see that even if inadvertently, they have treated the target of their demand as someone who should do as demanded simply because the demand has been issued.

In the case where the demander's belief is true, but unjustified or some defeater for the belief exists, the demand would still be disrespectful. This is because even if the person making the demand happens to have the true belief, this access to the truth is merely lucky. This is especially apparent when the belief is unjustified. Circumstances could have easily been different. The demand would likely have been made even if the world was different and the moral truth turned out to be different. This is so, even in the case where the belief is justified and true, but falls short of knowledge because defeaters for the belief exist. In that case, if the demander had learned of those defeaters, she could just as easily have correctly reasoned her way to not believing that her target should do as she is currently demanding. The demander's access to the truth is merely lucky. She is merely lucky that she did not encounter the defeater or even if she did encounter it, she is merely lucky that her impeccable reasoning led her to believe the truth. After all, the existence of a defeater for a belief implies that there is some consideration/evidence that, if added to one's own evidence, can make it rational to not believe the proposition in question. If there was no defeater, then disagreement would not be reasonable.

This is what makes moral demands in the bad case involve an attempt to impose will on someone else. It is that person's making of that demand and the social pressure or coercion that it generates that causes the target to comply. This disregards the fact that other people also have the capacity to respond to reasons and thereby work out for themselves what morality requires of them. Furthermore, to push someone around is to take oneself to have the authority to do so and further, to suppose that the target's deliberations about the matter would not matter regardless of the content of the deliberation. If making a demand on others is disrespectful when the demander's belief is false or when it is true but unjustified, it is also disrespectful when the belief is justified and true but a defeater for it exists, that is, when there is reasonable disagreement.

Of course, moral demands do not only function to push people around; they also play an important role in social coordination and in reminding each other what morality requires of them. Hence, in the good case, making a moral demand does not necessarily arrogate, to the maker of the demand, the authority to direct others' actions. Rather, the demander relies on the authority of morality itself to get the target to do as demanded. By demanding only what is publicly justifiable, we demand that someone do what, by her own lights, she has decisive moral reason to do. Even if the demand brings with it some social pressure or coercion, the person could still, in principle, be moved by her own faculty of reason to do as demanded.

If she happens to disagree with a publicly justifiable demand, then, either there is simply some mistake in reasoning that she is making or there is some crucial piece of evidence that she lacks. In the first instance, where there is a mistake in reasoning, demanding that she do as she ought is consistent with respecting her as, in this case, her belief genuinely is epistemically deficient in being irrational. Pointing out the truth is not in itself disrespectful. In addition, her status as a moral person is still respected in making the demand because the demander merely expects her to respond to her reasons properly and she is able to do so!

In the second instance, where she lacks crucial evidence, demanding that she do as she ought is still consistent with respecting her, as her beliefs are epistemically deficient in a different way: She is lacking a crucial piece of evidence that others have and the lack of which blocks her access to the moral truth. When someone has better access to the moral truth than someone else, correctly pointing that out is not disrespectful.

Let us sum up the argument in the paper so far. Bio‐restrictionism makes moral demands that are subject to reasonable disagreement, and religious bioethics is typically used to support such bio‐restrictionism in a public‐facing context. Also, the inclusion of religious bioethics in this context relies on a principle of respect, namely, that exclusion improperly denigrates religious belief and disrespects believers' status as moral agents. Yet, as we have just seen, making moral demands subject to reasonable disagreement improperly denigrates more permissive, but reasonable views like prime bio‐permissivism and disrespects reasonable liberals' status as moral agents. Therefore, the inclusion of religious bio‐restrictionism is exclusionary and disrespectful in a way that proponents of religious bioethics are committed to seeing as wrongful — proponents of religious bioethics thus have a duty to self‐exclude religious bio‐restrictionism in the public‐facing context.

## 4 Objections

Let us consider some objections to the view that I have argued for. First, one might, object that by my own lights, I am also advocating for the exclusion of certain reasonable views, namely, religious bio‐restrictionism, from the public‐facing context. Would this not count as disrespectful to them as well?

One initial reply is that I am arguably only making a third‐personal claim that those who endorse Sufficiency are also committed to self‐excluding their religious bio‐restrictionist views from the public square. People are free to reject Sufficiency. If they do, however, they cannot complain that the exclusion of religious bioethics disrespects their religious views or for that matter themselves.

One might find my thought that I am only making a third‐personal claim disingenuous. It does seem, after all, as if I am advocating for religious bio‐restrictionists to take themselves out of the public square. Even if this is right, my demand that they do so is not disrespectful. As I have just shown in this paper, religious bio‐restrictionists who advocate for the inclusion of their views in the public sphere are also committed to excluding their bio‐restrictionist views. Their disagreement with the conclusion is therefore unreasonable. Making second‐personal claims in the face of unreasonable disagreement is not disrespectful because I am merely demanding that they do as they ought to by their own lights. Neither am I implying that religious bio‐restrictionism is unreasonable as such, only their reluctance to self‐exclude that view.

Second, a related objection may be that religious bio‐restrictionists need not endorse Sufficiency. Instead, they could, like many proponents of nonreligious views, simply argue that their view should be reflected in law or ethics codes simply because it is correct.

My reply here is to note that anybody who is committed to respecting others as moral equals is also committed to self‐excluding views that are subject to reasonable disagreement. That is, they themselves are committed to believing that a view being correct is not sufficient to include it in ethics codes and the law. Moreover, denying moral equality is implausible. Let us consider two ways one might do so.

One way to deny that people are moral equals, in the requisite sense, is to think that it is possible that some moral agents are able to unilaterally create duties for other moral agents but not vice versa. After all, if all moral agents could, by making a demand create a duty to do as demanded, if one person demanded that another do something, the second person could just demand that the first retract her demand. This would effectively nullify any such ability to unilaterally create duties in others. If some such theory is true, it would seem completely ad hoc and awfully convenient that the group of people who was able to unilaterally create duties for others happened to be those who had one particular moral view.

Alternatively, denying moral equality might instead involve believing that some elect group of people had, independent of any empirical or testimonial evidence, superior access to the moral and religious truth. The point here is not that religious belief is necessarily irrational. After all, it may be reasonable for someone who has received much more testimonial evidence in favour of one religious doctrine rather than others to believe that doctrine instead of others. Even if she is aware that other people have received different testimonial evidence in favour of different religious doctrines, an agent might still maintain her belief, since she is intimately acquainted with her own testimonial evidence, but knows of conflicting testimonial evidence only second hand. For all she knows, if other people encountered her testimonial evidence in addition to their own, they would be required to endorse her religious doctrine instead of their own. This is still consistent with moral equality as one could believe that with sufficient empirical or testimonial evidence, everyone would have access to the moral truth. Denying moral equality requires one to go further and deny that everyone, even with sufficient evidence, would have access to the moral truth. Again, it seems ad hoc and awfully convenient that the elect group who has privileged access to the moral truth just happens to be one's own.

Once someone accepts that people are moral equals, then they are committed to thinking at least that a moral demand for someone to do something, ф, is permissible only if it is true that she ought to ф. After all, it is incoherent to say “you must ф, but ф‐ing is not obligatory”. As I have argued earlier, once we are committed to thinking that it would be wrong to demand that someone do what they are not obliged to do, we are committed to thinking that it would be wrong to demand that someone ф when someone could rationally disagree that they should ф.

At this point, one might counter that this would imply that on such a public justification requirement, very few laws could be permissibly enacted, since many of the norms underlying our laws are subject to reasonable disagreement. My reply here is that this is not an objection to the principle. Arguably, such a limited government would be desirable if the public justification requirement were indeed true (and publicly justifiable). Indeed, others have argued for classical liberal or libertarian conclusions on the basis of a public justification requirement [44].

I should take care to emphasise that like other principles that entail a limited role for religious views when it comes to the exercise of power [29], I am not arguing that people should be coerced into excluding their own views from the public setting. Rather, I am arguing that it is wrong for people to impose their bio‐restrictionist views on others. My view here is no worse than other more traditional views, according to which governments have a more limited remit of preventing certain harms or wrongs and should not concern itself with the health of our souls or our relationship with God, and so forth [45]. If it is wrong for governments to coercively impose certain requirements simply because it is required by some religions; it is also wrong for other bodies to impose such requirements via the exercise of power. It would also be odd for it to be morally permissible to advocate and try to get the government or other body to make such an imposition when such an imposition is morally wrong. Therefore, like my own view, on any traditional view where governments and coercive bodies have a limited remit, it is also wrong to advocate for the (wrongful) imposition of religious doctrine on others.

In fact, on my view, people are morally permitted to bring their theological or scriptural beliefs to bear if other people were imposing their views on them. They are also allowed to use religious reasons to get people to do what, according to every reasonable view, they ought to do. My view, unlike others' [29, 46], is silent on the ethics of excluding religious bioethics from journals and conferences.

## 5 Conclusion

Summing up, I have argued that anyone who is committed to the argument from adequacy is committed to excluding their own bio‐restrictionist views from the public‐facing context. We can readily extend this argument in three ways. First, we can expand the scope of who is committed to the public justification requirement for moral demands. Second, we can expand the scope of foundational commitments beyond religious ones. Any kind of philosophical doctrine, religious or otherwise, that grounds views more restrictive than prime bio‐permissivism must similarly be excluded. Third, we can expand the scope of excluded views beyond bio‐restrictionism. Any view more restrictive than prime bio‐permissivism will also violate the public justification requirement. Regardless of whether they accept Sufficiency, anybody who thinks that it is wrong to improperly denigrate reasonable views or to disrespect people qua their status as equal moral persons is committed to the public justification requirement and hence required to exclude any demands resulting from their own views that are subject to reasonable disagreement from the public sphere. If it turns out that everyone ought to endorse a duty to respect one another as moral equals, then everyone is committed to advancing only publicly justifiable views to the public‐facing context.

## Data Availability

Data sharing is not applicable to this article as no data sets were generated or analysed during the current study.

## Endnotes

1 One might think that restrictionist views, broadly conceived, do not arise only from social conservative concerns. They may also arise from more egalitarian concerns about inequality, exploitation and domination. This is true. However, while such views, arguably, are also subject to reasonable disagreement, proponents of left‐bio‐restrictionist views use a different argumentative strategy, which in turn would require a different response. This paper aims to address the argumentative strategy that is often employed by proponents of socially conservative viewpoints.

2 Indeed, there is no need to draw a sharp line. Strictly speaking, the argument that I make against bio‐restrictionism can also be made against any view that is more restrictive than the more permissive of the reasonable views. Any of these more restrictive views in the public‐facing context would involve imposing requirements that are subject to reasonable disagreement.

3 We might think of a piece of evidence as crucial or decisive if it is such that acquiring that piece of evidence overrides any other evidence that one might have for or against a given proposition.

4 Note that I intend this to mean someone, anywhere in the world, not necessarily from the same society or context, as the person issuing the demand. Hence, contra Jecker and colleagues (2024), I do not regard public justifiability as relativised to society. This would be so even if reasonableness of a view is relative to the society that one finds oneself in. Demands that would be justified only on the basis of an Islamic bioethics are no more publicly justifiable in Saudi Arabia or Malaysia than they would be in the United States or Singapore. After all, even if such demands are much more plausible in the Saudi context, someone in Iceland or Australia with very different evidence might still rationally disagree. Importantly, societies are not homogeneous and even in places with a strong religious culture, people could still reasonably disagree. Nonetheless, operationalising what is reasonable in a given society might still involve looking at what people actually believe [51].

5 I am specifying reasonable disagreement in this way because lots of people believe lots of things for very bad reasons. It does not follow that somebody could not still believe those same things for good reasons. On any plausible account of public justification liberalism, the mere fact that most people's religious and moral outlooks are not well reasoned out is not a good reason to violate their freedom of conscience. See. for instance, Muralidharan [52] and Leland and van Wietmarschen [53].

6 A mistake in reasoning involves either making an invalid inference or failing to make a valid inference from one's evidence about the subject matter under consideration [54].

7 Here, I remain silent about whether people with the same total body of evidence can rationally disagree.

8 See also Oswald [55] and Savulescu [56] for the distinction between scholarly work and activist bioethics. For a more pejorative label, “museum bioethics”, see Fan [27].

9 These may also be called Research Ethics Committees (REC) or Human Research Ethics Committees (HREC) in other jurisdictions.

10 Insofar as Cahill [4] and others endorse the role of religion in bioethics because it has an a‐rational influence on people's decisions, she endorses influencing behaviour by exercise of social power.

11 A common objection to arguments against religious bioethics is that the term religion is not adequately defined or perhaps in principle cannot be defined [42, 57]. As such, critics of religious bioethics may not be able to cleanly demarcate their target. It might seem as if my argument here is subject to the same criticism. However, it is not because my argument does not depend on religion being some natural kind rather than an artificial category. My argument is against bio‐restrictionism, religious or otherwise. It just turns out that for historical and sociological reasons, religious bio‐restrictionists have made an argument that is especially vulnerable to the objection that I make in this paper.

12 While there could be other moral foundations like concerns about hierarchy, outgroups, scepticism about progress and risk aversion that drive conservatism, more broadly conceived, purity/sacredness seem especially salient for conservatism with respect to bioethical questions.

13 See also Eberl [47] and Gómez‐Lobo and Keown [58] for the claim that “homo sapiens essentially has the capacity of personhood”. One is not rationally required to endorse such inflated metaphysical claims about particular species. By contrast, a thinner and hence less controversial claim is that most members of homo sapiens are persons.

14 See also Blackford and Schüklenk [29], who argue that terms like “dignity” and “vulnerability” are used in ways that smuggle in distinctly religious assumptions in order to argue for bio‐restrictionist conclusions.

15 Here, I refer to not only scholars but also religious personages (e.g. the local Imam, the Pope or the Dalai Lama), politicians and activists who comment publicly on bioethical matters.

16 Consider, for instance, that *Roe v. Wade* [49], which recognised a federal right to abortion, was weakened by the Planned *Parenthood v. Casey* [59] decision and finally overturned in the *Dobbs v. Jackson* decision [60]. *Dobbs* distances itself from the language of “potential” life found in Roe and Planned Parenthood and instead prefers the terms ‘unborn human beings’ or ‘prenatal life’, and argues that states have an interest in protecting such life. Arguably, this reflects a religious influence, where religious opponents of abortion believe that human life is morally significant from the moment of conception. In addition, Blackford and Schuklenk [29] have documented explicit efforts by Catholics like Margeret Somerville, Michael Cook and Steve Bannon to move policy in a more restrictionist direction. Explicitly religious arguments are relabelled with seemingly secular language of “dignity” in order to bring public policy in greater alignment with Catholic teaching. Consider also how the United States' President's Council on Bioethics, a body with significant political power, uses arguments about the purpose of human sexuality and procreation to argue against tinkering with the genes of one's offspring [48].

17 Other authors [61], who are also against religion in the public sphere, argue that we should use shared reasons for public bioethical discourse, but allow that people may reasonably disagree about how these reasons balance out against each other. By contrast, our position is that people may use unshared (even religious) reasons but may not make demands that people could reasonably disagree with.

18 Viana [50], Mayes [62], Anderson [63] and Hutchings [41] might seem to endorse a similar argument with respect to indigenous ethical frameworks, but the difference from religious bio‐restrictionists is that they are objecting to the imposition of liberal (but not prime bio‐permissivist) requirements on them, while bio‐restrictionists are advocating for the imposition of bio‐restrictionist views on those who reasonably disagree. The question of how the public justification requirement interacts with indigenous moral practices is beyond the scope of this paper.
